# The Importance of Consistent Global Forest Aboveground Biomass Product Validation

**DOI:** 10.1007/s10712-019-09538-8

**Published:** 2019-05-30

**Authors:** L. Duncanson, J. Armston, M. Disney, V. Avitabile, N. Barbier, K. Calders, S. Carter, J. Chave, M. Herold, T. W. Crowther, M. Falkowski, J. R. Kellner, N. Labrière, R. Lucas, N. MacBean, R. E. McRoberts, V. Meyer, E. Næsset, J. E. Nickeson, K. I. Paul, O. L. Phillips, M. Réjou-Méchain, M. Román, S. Roxburgh, S. Saatchi, D. Schepaschenko, K. Scipal, P. R. Siqueira, A. Whitehurst, M. Williams

**Affiliations:** 1 Department of Geographical Sciences, University of Maryland, College Park, 2181 Lefrak Hall, College Park, MD 20742 USA; 2 Department of Geography, University College London, Gower Street, London, WC1E 6BT UK; 3 European Commission, Joint Research Centre (JRC), Via E. Fermi 2749, 21027 Ispra, Italy; 4 AMAP, IRD, CIRAD, CNRS, INRA, Montpellier University, TA A51/PS2, 34398 Montpellier cedex 5, France; 5 CAVElab – Computational and Applied Vegetation Ecology, Ghent University, Room A2.089, Coupure Links 653, 9000 Ghent, Belgium; 6 Laboratory of Geo-Information Science and Remote Sensing, Wageningen University and Research, Droevendaalsesteeg 3, 6708 PB Wageningen, The Netherlands; 7 Laboratoire Evolution et Diversit. Biologique, UMR 5174, CNRS, Universit. Toulouse Paul Sabatier, 118 route de Narbonne, 31062 Toulouse cedex 9, France; 8 Institute of Integrative Biology, ETH Zürich, Univeritätstrasse 16, 8006 Zurich, Switzerland; 9 Department of Ecosystem Science and Sustainability, Colorado State University, Fort Collins, CO 80523 USA; 10 Institute at Brown for Environment and Society, Brown University, Providence, RI 02912 USA; 11 Department of Ecology and Evolutionary Biology, Brown University, Providence, RI 02912 USA; 12 Earth Observation and Ecosystem Dynamics Research Group, Department of Geography and Earth Sciences (DGES), Aberystwyth University, Aberystwyth, Wales SY23 3DB UK; 13 Department of Geography, Indiana University, 701 E. Kirkwood Ave., Bloomington, IN 47405 USA; 14 USDA Forest Service, Northern Research Station, Saint Paul, 1992 Folwell Ave, St Paul, MN 55108 USA; 15 Jet Propulsion Laboratory, California Institute of Technology, Pasadena, CA 91109 USA; 16 Faculty of Environmental Sciences and Natural Resource Management, Norwegian University of Life Sciences, NMBU, P.O. Box 5003, 1432 Ås, Norway; 17 NASA Goddard Space Flight Center/Science Systems and Applications Inc., 10210 Greenbelt Rd #600, Lanham, MD 20706 USA; 18 CSIRO Land and Water, GPO Box 1700, Canberra, ACT 2601 Australia; 19 School of Geography, University of Leeds, Leeds, LS2 9JT UK; 20 International Institute for Applied Systems Analysis, Schlossplatz 1, 2361 Laxenburg, Austria; 21 European Space Agency, ESTEC, Keplerlaan 1, 2201 AZ Noordwijk, The Netherlands; 22 Department of Electrical and Computer Engineering, 201 Marcus Hall, University of Massachusetts, 100 Natural Resources Road, Amherst, MA 01003 USA; 23 Arctic Slope Federal Technical Services, 7000 Muirkirk Meadows Dr #100, Laurel, MD 20707 USA; 24 School of GeoScience, University of Edinburgh, Drummond St, Edinburgh, EH8 9XP UK; 25 Earth from Space Institute, Universities Space Research Association, Columbia, MD USA

**Keywords:** Map validation, Biomass mapping, Reference data, Committee on Earth Observing Satellites, Remote sensing, Lidar

## Abstract

Several upcoming satellite missions have core science requirements to produce data for accurate forest aboveground biomass mapping. Largely because of these mission datasets, the number of available biomass products is expected to greatly increase over the coming decade. Despite the recognized importance of biomass mapping for a wide range of science, policy and management applications, there remains no community accepted standard for satellite-based biomass map validation. The Committee on Earth Observing Satellites (CEOS) is developing a protocol to fill this need in advance of the next generation of biomass-relevant satellites, and this paper presents a review of biomass validation practices from a CEOS perspective. We outline the wide range of anticipated user requirements for product accuracy assessment and provide recommendations for the validation of biomass products. These recommendations include the collection of new, high-quality in situ data and the use of airborne lidar biomass maps as tools toward transparent multi-resolution validation. Adoption of community-vetted validation standards and practices will facilitate the uptake of the next generation of biomass products.

## Introduction

Forest biomass has been recognized as a Global Climate Observing System (GCOS) Essential Climate Variable (ECV), a critical input to the United Nations’ Reducing Emissions from Deforestation and Degradation-plus (REDD +) program, and an important input to Earth system models (Herold et al. 2019). Spatially continuous maps of forest biomass are therefore important inputs for decreasing uncertainties in the global carbon cycle, particularly for areas where insufficient ground or airborne lidar data are available. Accurate biomass products are of great importance for forest management and climate mitigation. However, due to a previous dearth of satellite data specifically designed for producing accurate estimates of forest structure (Goetz et al. 2009) few global-scale forest biomass products are currently available, and the assessment of their accuracy is challenged by a lack of appropriate reference data. To overcome this critical carbon accounting gap, several upcoming Earth Observation (EO) missions will collect satellite data sensitive to forest structure and aboveground biomass, defined as the dry-weight of the live or dead woody component of aboveground vegetation. We anticipate a multitude of new global forest biomass products in the coming decade, but foresee challenges in intercomparison and validation across biomass products. These challenges have already been highlighted by several studies comparing the few existing continental or global-scale biomass products (Avitabile et al. 2016; Avitabile and Camia 2018; Baccini et al. 2012; Huang et al. 2015; Mitchard et al. 2013; Saatchi et al. 2011; Santoro et al. 2015; Thurner et al. 2014) and may hinder the effective adoption of biomass products for various policy, management and science applications.

A specific example of the importance of independent biomass product validation comes from comparisons of two widely known pantropical biomass maps (Baccini et al. 2012; Saatchi et al. 2011). By independent, we mean using reference data that were not included in the generation of products and ideally conducted by a third party. Despite having been produced from the same core satellite datasets (the Geoscience Laser Altimeter System [GLAS] and the Moderate-resolution Imaging Spectroradiometer [MODIS]), these maps differ substantially in several tropical areas (Avitabile et al. 2016; Mitchard et al. 2013, 2014) potentially because they employed different empirical modeling approaches, calibration datasets and extrapolation techniques. However, determination of the exact causes of discrepancies between these products, or indeed a determination of the more accurate product for a given application, is impossible without common approaches to independent validation. Aboveground biomass product validation is challenging, primarily because of the paucity of high-quality, publicly available and globally representative Fiducial Reference Measurements (FRM, https://earth.esa.int/web/sppa/activities/frm) with well-characterized uncertainties and challenges related to the fact that these reference data are not direct measurements but rather estimates based on tree-level allometric model predictions (Clark and Kellner 2012). Indeed, in the pantropical case, the map producers themselves had limited available validation datasets, and Baccini et al. (2012) and Saatchi et al. (2011) performed cross-validation of their mapped products using a subset of GLAS data that were deliberately left out of their biomass model training, rather than validating with an independent dataset. While Saatchi et al. (2011) conducted an error propagation for the final estimated uncertainties associated with their pantropical product and Baccini et al. (2012) reported confidence intervals on their estimates per continent, determination of the degree of accuracy of these products in geographic areas outside the calibration range, or at the various resolutions needed for policy implementation (Herold et al. 2019), was not possible. These products have been compared to the Intergovernmental Panel on Climate Change (IPCC) Tier 1 biomass estimates, following the 2006 IPCC Good Practices Guidelines (GPG), and although a composite of the two pantropical products was suitable to replace IPCC Tier 1 estimates when national inventories were not available, it was recommended that national estimates be favored over these remote sensing-based estimates, given the large disparities between the products and national inventories (Langner et al. 2014).

The issue of product validation will become even more pressing as the number of spaceborne datasets specifically designed to map ecosystem structure increases (e.g., from NASA’s GEDI, NASA/ISRO’s NISAR, ESA’s BIOMASS, JAXA’s ALOS-4) and approaches to biomass prediction using these data diversify. Previous biomass products have varied in terms of the spatial and temporal resolution, modeling approach, geographic scope of calibration data, scaling, error propagation and uncertainty reporting (Goetz et al. 2009; Huang et al. 2015; Mitchard et al. 2013).To effectively meet the goals of scientists and decision makers, the global change community requires well-tested validation approaches that are transparent and flexible (with respect to geographic scope and spatial resolution).

The Committee on Earth Observing Satellites (CEOS) is an international body that works to coordinate Earth Observation programs and data collected by space agencies. For nearly two decades, the CEOS Working Group on Calibration and Validation (WGCV) has had a subgroup specifically focused on Land Product Validation (LPV). In close coordination with CEOS member agencies, the LPV subgroup has recognized the need for good practices and protocols to guide biomass product validation in advance of the expected suite of upcoming biomass products. This LPV subgroup launched the biomass focus area in 2017 to help gather community support in developing a validation protocol for the products that will be generated from the upcoming biomass-related missions. This paper presents the conceptual development of the CEOS biomass protocol, reviews the specifications of the biomass products expected from new mission datasets and outlines the importance of biomass product validation for various biomass product user communities (e.g., for climate and carbon cycle modeling, policy applications and ecologists). We present the CEOS biomass protocol structure, including sources of errors inherent in biomass products, how these errors can be propagated and reported and which fiducial reference measurements are required to estimate product uncertainty. Finally, we discuss challenges envisioned by the authors in the implementation of the CEOS LPV validation protocol and its key recommendations.

## Background on Validation of Biomass Products

Biomass products have been generated across a range of spatial extents, from local and regional to global (Ahmed et al. 2013; Andersen et al. 2011; Avitabile et al. 2016; Baccini et al. 2008; Blackard et al. 2008; Boudreau et al. 2008; Duncanson et al. 2015a, b; Gregoire et al. 2011; Huang et al. 2015; Margolis et al. 2015; Neigh et al. 2013; Su et al. 2016). True biomass product validation requires the physical, destructive harvesting, drying, and weighing of trees. This is extremely difficult in practice, and typically undesirable or logistically impossible in many cases. As a result, validation of satellite-based AGB estimates relies on independent verification (i.e., inter-comparison of estimates made across scales with consistent and well-characterized uncertainties). For local biomass maps, validation is often conducted by the map producer, either through statistical cross-validation (i.e., using reference data not included in model calibration) or through comparison to independent data (e.g., from national forest inventories). These accuracy statistics are usually included in the publication of a biomass model, often expressed as a root mean squared error (RMSE), %RMSE and/or coefficient of determination (R^2^). For biomass maps, the accuracies are often expressed as a standard error or coefficient of variation. In local examples, while some researchers are diligent about reporting the specific validation methods used to generate their maps, others do not include these details, leaving map users unsure of whether the reported accuracies represent over-fitting to the calibration dataset, whether models exhibit systematic error, or whether model prediction residuals were heteroscedastic. Increasingly, these local biomass maps are used as reference data for larger area maps, and the errors associated with these reference products propagate in the final map.

In national, continental or global product validation, the challenge becomes greater, with local maps only representing a small proportion of the globe. Currently, there is no globally representative set of ground plots or local biomass maps sufficient for validation at the appropriate resolution of spaceborne products. The few global or continental-scale biomass products that have been released have relied on spaceborne estimates of biomass from NASA’s GLAS instrument for validation, often the same instrument used to generate the biomass product in the first place. In the case of Saatchi et al. (2011), the GLAS data used to validate the GLAS biomass model were not included when fitting the model, and the associated mapped products used a Monte Carlo error propagation to perform a product accuracy assessment. However, there are known issues with the GLAS biomass estimates in areas of high biomass or complicated terrain (Duncanson et al. 2010; Simard et al. 2011; Sun et al. 2008), and thus, any validation depending on GLAS data likely cannot capture the true errors associated with a GLAS-based product. Conversely, researchers have used forest inventory data to validate forest structure products (Simard et al. 2011), but these inventory datasets represent small sample of the landscape that may not be representative of the relatively coarse pixels (500 m–1 km) often associated with wide area biomass products.

We argue that it cannot be the sole responsibility of a dataset producer (be it a mission team or an independent academic) to conduct validation exercises over the range of scales necessary for all possible users; beyond the due diligence of transparently reporting the methods used to produce the map, and associated errors, an independent validation process is required. However, independent groups involved in validation exercises may vary in terms of the datasets available to them, the size of field inventory plots, availability of airborne lidar data, geographic scope of validation datasets, etc. Further, since some users may require different errors reporting, e.g., for IPCC compliance, national-scale estimates are desirable. Alternatively, ecosystem modelers will tend to require aggregated pixel-based errors. Additionally, users will inevitably be interested in different scales, be it local areas for forest management, national reporting efforts, continental carbon balance accounting, global ecosystem models, etc. To date, there is no clear guiding document focused on addressing the validation needs of many biomass product users and, for this reason, we present work toward a protocol that addresses this need.

## Earth Observation Missions for Biomass Mapping

NASA’s ICESat was launched in 2003, and its GLAS instrument collected global waveform lidar measurements over vegetation that were used to estimate forest height and structure until the last ICESat laser failed in 2009 (Abshire et al. 2005). GLAS data were not designed to study forest structure, but these data have nevertheless become popular for biomass mapping. These data are relatively sparse in spatial sampling, and each lidar footprint illuminated a nominally 65-m-diameter circle, which resulted in the mixing of reflected signal from ground and canopy surfaces, ultimately presenting challenges for estimating biomass in areas of high relief or structural complexity (Duncanson et al. 2010). Despite these challenges, many wide area biomass maps used GLAS data to map forest structure (e.g., Baccini et al. 2008; Margolis et al. 2015; Saatchi et al. 2011; Simard et al. 2011; Su et al. 2016).

Several upcoming missions (e.g., GEDI, BIOMASS, NISAR) should provide improved data for biomass mapping compared to those earlier sensors as they are designed with a primary science goal of mapping forest biomass. Official mission biomass products are expected from each of these missions, but because of the publicly available nature of these mission datasets we also expect a host of other new biomass products through data fusion and alternative algorithms, etc. We therefore anticipate the release of products with a range of spatial resolutions, geographic extents and temporal domains. Table 1 shows the expected resolution of core biomass products from upcoming spaceborne missions themselves, but fusion products will likely present both coarser and higher resolution maps.

**Table 1:** The expected upcoming biomass-relevant missions. Note that only NISAR, GEDI and BIOMASS are approved missions with a formal requirement for biomass mapping accuracy. Missions with no official biomass product are marked Not Applicable (NA), and missions that have yet to publish these requirements are listed at To Be Determined (TBD)

Mission	Funding agency	Launch Date (Expected or past)	Data type	Biomass product resolution	Geographic domain	Accuracy requirement
NISAR	NASA/ISRO	2021	L-band SAR	1 ha (< 100 Mg/ha)	Global	< 20% RMSE for < 100 Mg/ha
GEDI	NASA	December 5, 2018	1064 nm waveform lidar	1 km	ISS (± ~ 51.6°)	< 20% standard error for 80% of forested 1 km cells
BIOMASS	ESA	2022	P-band SAR	4 ha	Global except western Europe and North America	< 20% RMSE for AGB > 50 Mg/ha; 10 Mg/ha for AGB ≤ 50 Mg/ha
MOLI	JAXA	~ 2022	1064 nm waveform lidar	500 m	ISS (± ~ 51.6°)	TBD
SAOCOM1A	CONAE	October, 2018	L-band SAR	TBD	Global	TBD
ICESat-2	NASA	September 15, 2018	532 nm photon counting lidar	NA	Global	NA
ALOS-4	JAXA	2021	L-band SAR	TBD	Global	TBD
TanDEM-L	DLR	Planned ~ 2023	L-band SAR	1 ha	Global	20% accuracy or 20 Mg/ha

Many of these upcoming missions have specific biomass product accuracy requirements as part of the criteria by which mission success is determined (Table 1). Independent validation of these products at their nominal resolution would help demonstrate that requirements have been met. This is particularly useful if validation of biomass products from each mission or estimation approach can be conducted at the same set of sites, allowing direct comparisons between accuracies of each product in different forest types, environments, disturbance histories, etc. Comparisons between official mission products and other new biomass maps will also allow the community to appreciate the accuracy impacts of algorithmic improvements, data fusion approaches, etc., on product accuracy and ultimately reduce confusion and latency in the adoption of new biomass mapping approaches.

## The Importance of Validation for Biomass Product Users

While biomass product validation is important for product inter-comparison toward improved map products, and for demonstrating that missions have met their design requirements, it is also key for the uptake of biomass maps by many other communities. Here, we highlight several communities that will likely use the next generation of biomass products for a wide range of applications. Although this list is not exhaustive, it enables an exploration of many biomass product and validation needs. We briefly discuss considerations for biomass validation for policy applications, carbon accounting in ‘non-forest’ ecosystems (woodlands, savannas), belowground biomass estimation, and for modeling activities.

### Policy Applications

Aboveground biomass is an important input to several current and future policy initiatives and user groups, and all of these groups require information on biomass product accuracy, albeit in different ways. Uncertainty estimates are critical for achieving goals and commitments related to forest management, climate change and sustainable development. For example, the Paris Agreement on Climate Change requires transparent reporting on national greenhouse gases (GHG) emissions, and measuring, reporting and verification of forest-related mitigation activities, for which improvements in the quality of forest biomass and carbon stocks information is essential. Biomass products are also relevant for Goal 15 of the United Nations Sustainable Development Goals (SDGs) that includes improved forest carbon management, reduced deforestation, afforestation and reforestation at a global scale. Additional policy initiatives which will use the next generation of biomass products include the IPCC assessment processes and various sustainable land use initiatives as well as a wide range of local/subnational stakeholders. IPCC good practices guidelines are focused on national scale validation and reporting and recommend that (1) nations neither over- nor underestimate biomass so far as can be judged and (2) that uncertainties are reduced as far as practicable (IPCC, 2006, Volume 1, Chapter 1, Section 1.2). These recommendations are primarily geared at countries for their national reporting and suggest that biomass maps would be suitable as long as their predictions exhibit no systematic error at a national-scale and have lower errors than current estimation strategies. There is a wide range of considerations with respect to the stakeholders’ needs of biomass validation, including the spatial resolution, geographic extent and temporal domain of product validation, and the definition of forest or land type and biomass definition over which the validation is considered. Indeed, the use of biomass products by these policy communities is tightly linked to error reporting (Romijn et al. 2018). To suit this wide range of policy needs, independent product validation will need to be flexible enough to allow user-specified geographic scopes and spatial scales, including consistent and traceable error reporting at the national and/or regional levels, as well as by land cover class (Herold et al. 2019)

### Non-forest Vegetation Carbon Accounting

While non-forest vegetation contributes less than 20% of global biomass, they represent one half of terrestrial productivity and cover some 70% of the Earth’s land surface (Pan et al. 2013). These non-forest ecosystems include savannas, woodlands, chaparral and shrublands. They are ecologically distinct from forests both in terms of a) the climatic, edaphic and disturbance histories that prevent them from becoming intact forest and b) the plant form and function of trees growing outside of closed-canopy conditions. The functional and structural dissimilarities of these systems lead to special considerations for biomass product validation. For example, many of the allometric models developed for species typically found in forests do not apply to the same species growing in open canopy conditions. Therefore, error propagation through allometric models will not account for potential biases that may occur from applying the models outside of the range of environmental conditions for which they were calibrated. In some regions, e.g., northern Eurasia, issues of low canopy density have been accounted for by direct inclusion of tree density in biomass conversion factors (Schepaschenko et al. 2018). However, many regions lack the necessary data to perform such corrections. We therefore recommend more attention to the development of new allometric biomass models in non-forest regions.

Non-forest vegetation also presents challenges for validation when many biomass products will be designed specifically for ecosystems classified as forests. Global biomass map producers may even remove many non-forest classes from their mapping domains or may adopt different forest–non-forest masks at different resolutions which will complicate product inter-comparisons. Additionally, due to land use and land cover change dynamics, ‘non-forest’ vegetation at the boundaries of intact forest changes over time, where disturbance and recovery may lead to pixels being somewhat arbitrarily classified as forest or ‘non-forest’ through time. This would influence the magnitudes of forest biomass stocks and fluxes more than the actual carbon flux associated with disturbance agents. Regardless of the methods used to define boundaries between ‘forest’ and ‘non-forest,’ it is important to be able to track biomass dynamics across these boundaries consistently and without bias (not systematically over or underestimating forest area) as far as possible. In an independent validation process, a clear indication of which forest mask was applied should be clearly indicated, and biomass products that include ‘non-forest’ biomass should be validated specifically in these other ecosystems rather than merging ‘non-forest’ accuracies with non-ecosystem specific error reporting.

### Belowground Biomass

The CEOS LPV biomass validation protocol is focused on aboveground biomass. However, we also recognize the importance of belowground biomass and therefore will provide a summary of both how soil scientists will use aboveground biomass products and also the state of the art of belowground biomass validation techniques. Global soil carbon has been estimated to be in excess of 2000 Pg in the top 1 m of soil, much of that outside of forests (Batjes 1996). These carbon pools include both root biomass, which is typically related to aboveground stocks, while Soil Organic Carbon (SOC) is often not correlated to aboveground structure and thus cannot be estimated from space.

There is a strong need to link belowground stock and flux measurements with the aboveground focus of EO missions and forest inventories. Current practices for estimating belowground biomass include localized field work (e.g., digging pits) or applying indirect (remote sensing) methods such as ground penetrating radar to detect a range of soil characteristics including soil depth (Wollschläger et al. 2010), carbon storage (Hruska et al. 1999) or root quantities and distributions (Comas et al. 2017). These field-based methods are not applicable to wide area mapping and can only serve to calibrate or validate spaceborne estimates of belowground biomass. When field data are not available, typically simple root-to-shoot ratios are applied to estimate belowground biomass, although these are known to be poorly constrained in many systems and require much more attention to develop globally representative models (Mokany et al. 2006). Spatially continuous approaches use spectral data to map forest type and function and assume relationships between vegetation reflectance and belowground biomass (e.g., Fisher et al. 2016; Hengl et al. 2014, 2017). Despite the considerable importance of belowground biomass to the global carbon cycle, and the link between aboveground and belowground carbon stocks, this field is relatively less mature in terms of product validation. Although the CEOS LPV biomass protocol will only touch on some of the considerations of belowground validation, we expect a more specific assessment regarding belowground biomass validation to follow as aboveground biomass good practices continue to mature.

### Modeling Community Needs

Reliable estimates of biomass, particularly through time, present opportunities to reduce current knowledge gaps in carbon cycle modeling. For example, the allocation of carbon to different plant tissues, as well as the timescale of turnover of these tissues, remains one of the key uncertainties in vegetation modeling and projections of the terrestrial carbon sink. Many different types of models would benefit from biomass products and thus validation activities, but the nature of the models will influence their validation requirements. For example, carbon-pool models operating at a specific spatial resolution (e.g., 1 ha, or 1 degree postings) would use pixel-based predictions of aboveground biomass (and error) as inputs. Conversely, cohort models may require resolving a biomass map into classifications of land cover and consider error as an average for a given land cover class (e.g., different forest types). Similarly, forestry and economic land use models require administrative level error reporting (e.g., at national extents). Finally, individual-based models such as gap models operate within larger forest patches and thus will likely require biomass (and associated height) errors at finer resolution and smaller geographic extent than the broader-scale model types. To provide modeling groups with informative biomass product errors, error should be reported across multiple resolutions and spatial extents. In addition, producers of biomass maps should be encouraged to document and quantify errors associated with not only the biomass products, but also from the input data (e.g., height error from lidar) and empirical models as they will ultimately inform modeling results and improve the integration of biomass estimates into modeling activities. This recommendation is in addition to the requirement for validation over multiple spatial scales and geographic extents, which is also important to a range of other communities and applications.

## CEOS LPV Validation Protocol Structure

There are clear overlaps between the needs of several of the communities highlighted above. Flexibility of validation in terms of spatial scale, geographic scope, error reporting, forest definition selection and considerations of both biomass stocks and fluxes are recurring themes when discussing user community requirements (e.g., policy, modeling, soil science, dryland ecology). Therefore, we aim to develop a biomass validation protocol with the flexibility to account for the breadth of user needs. The goal of the CEOS WGCV LPV biomass protocol is to provide a good practices guidance document with recommendations for how to conduct independent biomass product validation. Here, we present what we see as the primary considerations for data collection and validation design in this framework. We also highlight some challenges biomass map producers face with respect to enabling reliable, consistent and transparent product validation.

### Reference Datasets

Any validation depends on the availability of high-quality reference datasets that are independent from the data used to generate an AGB product. Large area AGB datasets used for validation are usually from either forest inventories, forest monitoring plots or local airborne lidar biomass maps, all of which include error in their estimates of biomass. Forest inventory datasets typically consist of large numbers of small, geographically dispersed plots designed to sample across a defined geographic extent or region, often national or local, and are typically updated at some systematic time interval. Conversely, forest monitoring plots tend to be fewer in number but larger in area and collect a broader range of environmental datasets, with individual trees carefully identified and measured over several years to track biological and ecological trends as well as estimation of biomass. Although arguably more useful for pixel-level comparison to AGB maps, these plots typically do not represent probability samples. Finally, airborne lidar biomass maps provide wall-to-wall coverage that can be used to estimate biomass at multiple spatial resolutions. These lidar maps are usually calibrated with local field measurements, either from inventory plots, monitoring plots or field data often collected specifically as part of the lidar collection. These include (by definition) more error at the pixel-level than the field plots used to calibrate them.

None of these three types of reference data represent actual measurements of forest biomass, which is an important distinction from many of the other GCOS ECVs (e.g., Land Surface Temperature) where validation is performed using fiducial reference measurements. Fiducial reference measurements or FRM are a specific kind of reference data, traceable to International System of Units (SI) working, developed with national metrology institutes and produced together with documented uncertainties. As mentioned above, direct aboveground biomass measurements can only be collected through the destructive harvest and weighing of trees, which is of course inherently inappropriate for monitoring plots and logistically unfeasible for inventory plots (Clark and Kellner 2012). Instead of directly measuring biomass, field estimates rely on one of two methods—the measurements of tree and forest plot data and application of allometric models (e.g., Chave et al. 2014a, b; Jenkins et al. 2003), or the use of a Terrestrial Laser Scanning (TLS) instrument (Calders et al. 2015) combined with estimated wood density. In the former, individual tree biomass is predicted through the application of empirical models of relationships between measurable tree attributes (e.g., species identity, stem diameter, height) to aboveground biomass. These allometric size-to-mass models are estimated from destructively sampling trees and often from small samples (sometimes fewer than 30 trees) with a prevalence of small-size trees (Duncanson et al. 2015a, b). Although it is recognized that these models often exhibit systematic prediction errors (Ahmed et al. 2013; Chave et al. 2014b; Gonzalez de Tanago et al. 2018) and that these errors increase with tree size, they are currently the most practical approach for estimating field biomass in a cost-effective fashion. Indeed, statistical packages are now available to both estimate and propagate errors in field biomass estimates to forest plot levels (Réjou-Méchain et al. 2017).

The second (and more recent) approach for estimating field plot volume (and biomass where wood density information is available) is to use TLS instruments to scan forest plots with a ground-based lidar instrument and reconstruct detailed 3D scenes of a forest plot that include individual tree woody materials (Disney et al. 2018; Newnham et al. 2015). TLS datasets, when processed to predict plot-level tree volumes, may produce models whose predictions are more accurate relative to allometric biomass predictions, largely because they represent the full tree size distribution that may be underrepresented in allometric models (Calders et al. 2015; Gonzalez de Tanago et al. 2018). Although TLS data may be preferred when available, the relatively large cost and effort required to collect high-quality TLS data from remote forests, and the current challenges in data processing (including large computational demands and relatively immature software availability, (Newnham et al. 2015; Trochta et al. 2017), mean that these measurements are typically only available over a relatively small set of forest monitoring plots (Disney et al. 2018). It should also be noted that TLS data require wood density information from tree species identification and thus will never fully replace field surveys but add more complete structure measurements of forest plots over manual diameter and height measurements. Attention is required to ensure that TLS data do not overestimate the woody volume of hollow trees, and we recommend further research into TLS allometric model development and correction factors for varying wood density and hollow trees. Instead, TLS data may be most useful to check and re-calibrate allometric models with larger sample sizes and across the full range of tree sizes, thereby reducing underestimates of biomass, particularly for large trees. Considering that ~ 50% of landscape-scale forest biomass may be stored in a few large trees, particularly in the tropics, reducing errors and biases related to large tree biomass stocks is particularly important (Lutz et al. 2012, 2018).

### Geolocation, Temporal and Spatial Scale

Once plot-level biomass has been predicted, spaceborne products must be spatially linked to these reference plots. This process adds error to validation efforts because spaceborne and field datasets have inherent geolocation errors associated with them. This is especially true for field measurements where large errors may occur due to important GNSS multipathing effects in forest ecosystems (Barton and Johnson 2004). Even with the use of high-grade GNSS devices, some unfavorable conditions due to the number and position of satellites or to local forest structure and topography may generate large geolocation errors (~ 10 m). Increasing the number of GNSS measurements in space and time (e.g., > 15 measurements per plot) would be one way to achieve an accurate location, as would the use of a pole-mounted GPS antenna. Another major problem is that field plots sizes and shapes usually differ from the sizes and/or shapes of map units characterizing spaceborne biomass products. This is particularly problematic when attempting to validate relatively coarse (500 m–1 km) biomass products with smaller forest inventory plots. For instance, Réjou-Méchain et al. (2014) found that large errors (typically 30%) can be introduced to the calibration/validation procedure from using small forest plots (less than 0.25 ha) in this manner. Importantly, they demonstrated that this error induces bias into the estimator of RS model parameters and, in turn, systematic error in RS model predictions (Réjou-Méchain et al. 2014).

Small plots are also challenging even when validating finer-resolution (e.g., 30 m) remote sensing products for several reasons. At such resolutions, a small spatial or configuration mismatch between the field and the EO product may generate important errors due to the large local heterogeneity of forest biomass. Further, in areas with large tree crowns, satellite data for a plot may correspond to a part of a crown for a tree whose stem may not fall within the plot (Mascaro et al. 2011). As plot size increases, the relative errors associated with so-called edge effects and geolocation decrease. Thus, higher correlations are typically found between field plots and remote sensing datasets when large (e.g., ≥ 1 ha) plots are used (Huang et al. 2013; Zolkos et al. 2013). One solution to the small plot problem is to implement a screening procedure to remove plots that are not representative of the larger pixels and to obtain a subset that also maintains the frequency distribution (i.e., histogram) of the original dataset (Avitabile and Camia 2018).

Where this screening procedure is not practicable, and in regions with relatively large errors associated with plot size, (e.g., large crowns, greater geolocation uncertainties), such as in the tropics, we recommend the use of relatively large field plots to calibrate models based on satellite datasets, where possible. Additionally, we recommend validating with plots that are the same spatial resolution as satellite-based products to avoid issues from dilution bias (Réjou-Méchain et al. 2014). However, relying on large plots may reduce the number of plots that can be measured for validating a biomass product. Thus, there is a trade-off between the use of large plots and the need for capturing environmental gradients that drive variability in forest structure, and/or errors in spaceborne data acquisitions (e.g., canopy density, topography, soil moisture). Although errors from co-registration, temporal differences and edge effects all decrease with increasing plot size, typically sampling errors will increase with plot size because the sample size (number of plots) will decrease, assuming constant sampling design budget constraints.

The temporal discrepancies between field plots, airborne datasets and spaceborne datasets are also critical for product validation. Different forest ecosystems will change at different rates, due to inherent differences in productivity, as well as stochastic disturbances, ecosystem recovery and natural demographic shifts. The rate of change of a given ecosystem therefore determines the importance of collecting validation data that is temporally coincident with satellite biomass products. In slow growing areas with little disturbance, validation data from up to ~ 5 years before or after satellite data collection may be suitable, but in more productive ecosystems and forests that are degraded or regenerating, or have undergone disturbance validation data will require more frequent updating. Assuming plots and satellite data can be precisely geo-referenced, one solution to determining whether validation data remains useful is to use a Landsat-based change detection dataset, e.g., Harris et al. (2012), Kennedy et al. (2010), to flag pixels that have undergone significant change in the time elapsed between field/airborne and satellite data acquisitions. Alternative approaches capable of resolving finer-scale disturbances (e.g., individual tree fall) may also emerge due to the increased availability of high spatial and temporal optical datasets, such as from Planet. Admittedly these approaches will likely not resolve disturbances in the understory, and thus, we recommend that where possible validation data should be as close in time as practicable to satellite data collection. Older data should only be considered when they represent geographic gaps, and local knowledge and spaceborne optical sensors suggest no significant change in forest biomass or cover.

### Scaling from Ground Plots to Airborne Lidar Maps

A strategy to minimize the trade-off between plot size and representativeness can be the use of locally calibrated airborne lidar biomass maps to scale between field plots and satellite datasets. These maps are increasingly used as reference data for spaceborne products, primarily because they cover a much larger spatial extent than field plots and thus greatly increase the area over which in situ information is mapped if their estimates are adequately calibrated across environmental gradients with respect to forest structural and species variability. Further, provided data are acquired with acceptable sensor and survey configurations, lidar data can easily be aggregated to multiple resolutions and thus a single airborne lidar campaign can serve to validate multiple biomass products with different spatial resolutions.

It is generally recommended to generate the airborne lidar maps at large (e.g., 0.25 ha or larger) pixel sizes in the tropics because errors related to geolocation and edge effects are reduced as plot sizes increase. This is also true of many temperate systems (e.g., Huang et al. 2013), but small plots may be appropriate in many temperate and boreal forests, depending on the demographic structure of the forest and quality of plot geolocation. In the case of the tropics, for example, if one calibrates a 25-m lidar map with 25 m plots, and aggregates predictions to 1 ha, errors will likely be greater than if calibrating a 1-ha lidar map with 1 ha plots (Labriere et al. 2018). If errors are propagated from ground plots to local lidar maps, finer-resolution lidar models and maps are also appropriate, e.g., McRoberts et al. (2018).

### Error Characterization and Propagation

A key aspect of robust product validation relates to error characterization and propagation. We recommend the transparent reporting of which errors are considered in a validation process, and what error propagation technique is used to produce error estimates on the reference datasets used. It is essential to consider that even the highest quality field data and airborne lidar maps contain errors. The CEOS LPV biomass protocol will include a thorough discussion outlining the types of errors that should be considered (many of which are discussed above) and present three basic methods of error propagation. These recommendations are in line with the IPCC Good Practice Guidelines, which are discussed in (McRoberts et al. 2019). We define ‘error’ as a deviation from field-estimated AGB values, and ‘uncertainty’ as the range of values within which the field value lies, with some reported level of confidence. The quantification and propagation of errors results in a reported product uncertainty, and there are several different approaches adopted at reporting product uncertainty. Validation with independent data will quantify error (deviation from reference data) with associated uncertainty in that error (from uncertainties in the reference datasets themselves). In the case of a product generated with careful attention to error, we would expect that the outcome of the validation would be an error with an associated uncertainty that is within the range of uncertainty reported by the map producer.

There are several different approaches to quantifying uncertainty in a product (both by the map producers and the map validators). Design-based uncertainty estimation is appropriate where probability-based validation datasets are available and collected specifically to sample the population of interest. These datasets are typically only available for forest inventory plots, which present challenges related to small plot sizes, as discussed above. Also, such inventories rarely exist in tropical forest environments, and the effort required to establish them is prohibitive. A second form of uncertainty estimation is in model-based inference, where the validation datasets are often not probability samples of a forest, but a network of opportunistically collected plots (e.g., collected to calibrate an airborne lidar campaign). In model-based inference, the uncertainties are based on the effects of sampling variability as reflected in the data used to construct the models and residual variability of sample observations around their model predictions. A third technique for calculating errors and associated uncertainties is from hybrid inference, which uses a combination of design-based and model-based inference. McRoberts et al. (2019) present a statistically rigorous test based of hybrid inference that can be used to assess the validation of a coarse resolution global biomass map using finer-resolution, higher-quality local biomass maps. Hybrid inference thus provides a possible statistical solution for validation using airborne lidar biomass maps.

### Validation and Reporting

Once a reference dataset has been collected and scaled to match the resolution of the biomass product in question and the associated errors have been characterized, quantified and propagated, the actual product validation is conducted. The reported accuracies (defined as a lack of error) and uncertainties for the product in question depend on the user requirements for the biomass product, as discussed in Sect. 4. Ecosystem models running at a global scale may need the average coefficient of variation, or confidence interval, per 1-degree grid cell. However, models running at a local or regional scale might require pixel level maximum or minimum estimates of biomass (e.g., from a confidence interval). Conversely, REDD + activities will likely focus on national-level biomass reporting and thus focus on the estimated errors (e.g., biases from the truth) and associated uncertainties at an aggregated national scale. We expect most users will aggregate uncertainty assessments to the appropriate scale for their application. As biomass product accuracies and uncertainties are likely to vary as a function of forest structure and geography (e.g., differing between tropical and temperate systems, mature and recovering forests), we recommend reporting at a variety of scales. For example, if a reference dataset is a geographically dispersed set of linked field and airborne lidar datasets, uncertainties should be reported at global, continental and ecoregion scales. It is unlikely that such a dataset would be representative at a national scale, but according to IPCC good practices the standard error per ecoregion could be used by a country in tandem with national and local data or available ecoregion maps to use the biomass product toward improved REDD + reporting (https://reddcompass.org/). The CEOS LPV biomass protocol will include specific recommendations for reference datasets and error and uncertainty reporting, but it should be stressed that any independent validation of AGB maps should be sufficiently flexible enough to allow errors and uncertainties to be reported at a variety of spatial resolutions, geographical and thematic scopes. It is important to note that we do not include recommendations for product harmonization or inter-comparison and instead suggest comparing products to fiducial reference datasets in order to avoid confusion when discrepancies are found among biomass products.

## Implementation Considerations

Independent validation requires the availability of independent reference datasets. Ideal reference datasets can be thought of as ‘super sites’ with highly accurate field measurements (Chave et al. 2019), TLS and airborne or drone lidar data (Kellner et al. 2019) (i.e., sites that have been surveyed in a manner allowing aggregation of lidar data at multiple resolutions and collected at the same time as the satellite data used to construct a given biomass product). To conduct a thorough product validation, these sites would have to be distributed across the geographic scope of interest and capture the important environmental gradients driving the relationships between biomass and error, such as disturbance history, climatic and topographic gradients, etc. However, these sites are relatively rare, particularly in the tropics, where much of the aboveground biomass is stored, and where they do exist they are costly to maintain and repeat survey. Including reference data with larger geographic and temporal coverage is, thus, important for the purpose of capturing more regional variation in biomass estimates and associated uncertainties. Such data can be provided by forest plot observation networks, research plots and forest inventories. Setting up and maintaining a permanent plot network of biomass supersites is key to conducting meaningful validation activities.

**Fig. 1:**
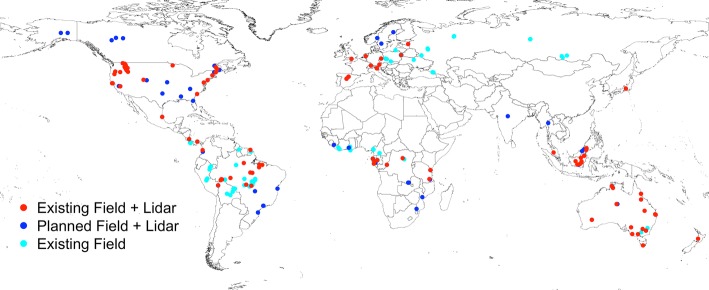
Existing or planned field and airborne lidar datasets for use in spaceborne mission biomass model fitting or product validation. The red points have been compiled by the NASA GEDI team and are used in their biomass model development. The blue points, compiled by the NISAR team, are planned sites for both model development and product validation. The turquoise sites, compiled by the Forest Observation System (FOS, Chave et al. 2019), represent high-quality standardized field estimates of biomass

Multiple upcoming missions with biomass mapping requirements also have requirements for product and/or algorithm validation, and these activities include budgets for new data acquisitions. These mission teams (namely GEDI, NISAR, ESA BIOMASS and ICESat-2) are actively working together to streamline calibration and validation data collections and maximize the geographic coverage of validation data roughly representative of forest conditions in the year 2020 (Fig. 1). However, these mission activities will not sufficiently cover many geographic domains. Of particular note is the lack of sites in continental Asia and expanding collaborations with other CEOS member agencies who may be able to help expand data collection activities is highly desirable.

It is also important to note that enabling a transparent and reproducible map validation requires open data. Open data allow transparency to validation activities, and reproducibility of results which we feel is essential for the wide adoption of the CEOS LPV recommendations for biomass product validation. This is however a complicated issue, as balance must be met between credit for the cost, skills and labor required for field data collection and curation, and the importance of open data for the scientific process. The FOS system has struck compromises between open data and maintaining census data privacy by publicly releasing only plot estimates of biomass with consistency propagated error (see Chave et al. 2019), and in situ data collected through funds from NASA and ESA are open by definition; hopefully more space agencies and plot networks will follow suit. A more equitable solution to this problem can be found if adequate long-term funding is provided to plot networks to ensure not simply that tree-by-tree data are collected and processed, but that the (mostly tropical) people doing this are adequately rewarded, trained and respected for their skills and efforts. In a more equal and secure environment, we argue that open publication of datasets will become a natural expectation.

Once global field and airborne lidar biomass reference datasets are compiled, a final challenge lies in actually conducting an independent validation and reporting the results. Ideally, fiducial reference data will be freely available so that any organization or researcher could conduct a transparent product validation. It is desirable that online tools become available that enable not only data access, but also error characterization and propagation in the generation of reference datasets such as airborne lidar maps. Existing tools such as Google Earth Engine provide unparalleled capabilities and potential for user-friendly product generation, but have yet to develop the necessary tools for validating products. Indeed, this requires a relatively specific set of tools and we recommend the development of an independent toolkit to perform biomass product validation following CEOS WGCV LPV biomass protocol recommendations. The BIOMASS package (Réjou-Méchain et al. 2017) is already widely used by many researchers and mission teams to propagate errors from individual tree estimates to field plots in the tropics, but comparable tools are not yet available to expand this to airborne lidar maps, particularly at multiple resolutions as is needed by the breadth of expected biomass product users. The new ESA/NASA Multi-Mission Algorithm and Analysis Platform (MAAP) is an activity focused on developing cloud-based tools for biomass product generation from active remote sensing datasets (primarily from GEDI, NISAR, and the ESA BIOMASS Mission). The MAAP is currently under development and is anticipated to be a useful platform for both housing and implementing biomass validation data. The Forest Observation System (FOS, https://forest-observation-system.net/) is also collating field validation datasets with preprocessed plot data that include propagated errors, and it is currently expanding to include airborne lidar-based biomass maps. The ESA Climate Change Initiative (CCI) Biomass Project (building on the ESA GlobBiomass project), similarly plans to support these activities by encouraging collaboration and cooperation in the application of the CEOS LPV protocol through the Global Plant Biomass Facility. The intention is to encourage and facilitate unified approaches to the collection of forest inventory data (including in near real time) and democratize access to in situ biomass estimates. All of these new activities illustrate the existing gap in biomass product tools and present exciting potential opportunities for implementing the CEOS LPV biomass protocol.

## Summary and Next Steps

The CEOS WGCV LPV biomass protocol document (to be published in 2019) will cover a thorough range of validation considerations, including the collection of new reference datasets, and recommendations for using airborne lidar biomass maps as tools for scaling between field and satellite data. The protocol will also consider the characterization, propagation and ultimately reporting of errors throughout the validation process, as well as the existing limitations for the implementation of product validation. The protocol document is intended to guide both biomass map producers and users toward consistent interpretation of product errors, with the ultimate goals of reducing confusion about which biomass products are more accurate or appropriate for a given application and increasing the suitability of new data collections for product validation. Considering the time and cost-intensive nature of collecting high-quality in situ and airborne datasets, a document that can facilitate, to the degree possible, standardization of data collection and methods for product validation will hopefully help streamline new acquisition plans across different agencies, and allow for easier interpretation of mapped products and errors. Equally, establishing and maintaining permanent reference sites for biomass, including the ongoing acquisition of field and airborne data, and where in situ partners are fully involved, will be key for conducting meaningful and sustainable biomass product validation. We are entering an exciting era in Earth Observation, with a wealth of new datasets from forthcoming missions leading to the anticipation of many new biomass products. The societal and scientific needs for accurate biomass products are clear—and with the promotion of transparent independent product validation at the range of scales required by data users we expect the next generation of mission datasets to successfully fill what has been a limiting knowledge gap in the global carbon cycle.
